# Investigation of a Hypoxia-Immune-Related Microenvironment Gene Signature and Prediction Model for Idiopathic Pulmonary Fibrosis

**DOI:** 10.3389/fimmu.2021.629854

**Published:** 2021-06-14

**Authors:** Xinyu Li, Haozheng Cai, Yufeng Cai, Quyan Zhang, Yinghe Ding, Quan Zhuang

**Affiliations:** ^1^ Transplantation Center, The 3rd Xiangya Hospital, Central South University, Changsha, China; ^2^ Xiangya School of Medicine, Central South University, Changsha, China; ^3^ School of Life Science, Central South University, Changsha, China; ^4^ Research Center of National Health Ministry on Transplantation Medicine, Changsha, China

**Keywords:** idiopathic pulmonary fibrosis, microenvironment, hypoxia, immune, prognosis

## Abstract

**Background:**

There is growing evidence found that the role of hypoxia and immune status in idiopathic pulmonary fibrosis (IPF). However, there are few studies about the role of hypoxia and immune status in the lung milieu in the prognosis of IPF. This study aimed to develop a hypoxia-immune-related prediction model for the prognosis of IPF.

**Methods:**

Hypoxia and immune status were estimated with microarray data of a discovery cohort from the GEO database using UMAP and ESTIMATE algorithms respectively. The Cox regression model with the LASSO method was used for identifying prognostic genes and developing hypoxia-immune-related genes. Cibersort was used to evaluate the difference of 22 kinds of immune cell infiltration. Three independent validation cohorts from GEO database were used for external validation. Peripheral blood mononuclear cell (PBMC) and bronchoalveolar lavage fluid (BALF) were collected to be tested by Quantitative reverse transcriptase-PCR (qRT-PCR) and flow cytometry from 22 clinical samples, including 13 healthy controls, six patients with non-fibrotic pneumonia and three patients with pulmonary fibrosis.

**Results:**

Hypoxia and immune status were significantly associated with the prognosis of IPF patients. High hypoxia and high immune status were identified as risk factors for overall survival. CD8+ T cell, activated CD4+ memory T cell, NK cell, activated mast cell, M1 and M0 macrophages were identified as key immune cells in hypoxia-immune-related microenvironment. A prediction model for IPF prognosis was established based on the hypoxia-immune-related one protective and nine risk DEGs. In the independent validation cohorts, the prognostic prediction model performed the significant applicability in peripheral whole blood, peripheral blood mononuclear cell, and lung tissue of IPF patients. The preliminary clinical specimen validation suggested the reliability of most conclusions.

**Conclusions:**

The hypoxia-immune-based prediction model for the prognosis of IPF provides a new idea for prognosis and treatment.

## Introduction

Idiopathic pulmonary fibrosis (IPF) is a chronic, progressive interstitial lung disease. The prognosis of patients with IPF is poor, with a median survival of 3 to 5 years (1). Several prognostic staging systems for IPF have been established by clinical and physiologic variables (2–5). Biomarkers in peripheral blood are also evaluated as a tool for prognosis (6–8). A recent study also revealed the role of genetic variability in the survival of IPF (9). In addition, molecular markers of IPF patients could also be identified by bronchoalveolar lavage (BAL) cells, and the collection of BAL cells is non-invasive compared with lung biopsy (10). However, very little is known whether molecular events in the lung milieu are predictive of outcome in IPF. Considering accurate diagnosis and personalized treatment, there is still a critical need for a way to predict the progression of IPF.

As a chronic lung disorder, the central processes in IPF are inflammation and fibrosis (11). Immune dysregulation has been considered as a promoting factor in the development of IPF, including several biomarkers associated with the prognosis of IPF (12). Inflammatory cytokines released by immune cells may activate fibroblasts, connective tissue cell proliferation, angiogenesis (11). Furthermore, hypoxia is common in the process of fibrosis in many diseases (13, 14). Excessive collagen synthesized by fibroblasts deteriorate oxygen supply and accelerate the pathological process. Studies showed the relationship between immune response and hypoxia and lung function (15). However, the underlying mechanisms have not been discussed.

With a series of genetic and bioinformatics analyses, we associated immune status with hypoxia and explore its value for the prognosis of patients with IPF. Here, we developed a hypoxia-immune-related prediction model for the prognosis of IPF, intended to provide novel ideas for accurate diagnosis and treatment at the gene level. Better knowledge of the oxygen balance control and the immune regulation involved is important to advance the development of IPF.

## Materials and Methods

### Patient Cohort and Data Preparation

The discovery cohort of the study contained 176 IPF patients from the Gene Expression Omnibus (GEO, available at: https://www.ncbi.nlm.nih.gov/geo/) database (GSE70866). The tissue source of sequencing samples is the patients’ BAL cells. The microarray data of GSE70866 was based on GPL14550 Platform (Agilent-028004 SurePrint G3 Human GE 8x60K Microarray, Agilent Technologies) and GPL17077 Platform (Agilent-039494 SurePrint G3 Human GE v2 8 × 60K Microarray, Agilent Technologies), including 176 IPF patients’ BAL cells. Three validation cohorts were used for external validations (GSE93606, GSE28221, and GSE32537) to examine the predictive effect of the prediction method. The microarray data of GSE93606 was based on GPL11532 Platforms (Affymetrix Human Gene 1.1 ST Array, Affymetrix, Santa Clara, CA, USA), including 57 IPF patients’ peripheral whole blood. The microarray data of GSE28221 was based on GPL5175 Platforms (Affymetrix Human Exon 1.0 ST Array, Affymetrix, Santa Clara, CA, USA) and GPL6480 Platforms (Agilent-014850 Whole Human Genome Microarray 4x44K G4112F, Agilent Technologies), including 120 IPF patients’ peripheral blood mononuclear cell. The microarray data of GSE32537 based on GPL6244 (Affymetrix Human Gene 1.0 ST Array, Affymetrix, Santa Clara, CA, USA) included 119 lung tissues with IPF. The batch effect was eliminated by sva package, which contains functions for identifying and building surrogate variables for high-dimensional data sets.

All procedures of this study complied with the protocol. For analyses of data from a public database, approval and informed consent from the local ethics committee were not required.

### Identification of Hypoxia Status and Hypoxia-Related DEGs

To identify the hypoxia status, a non-linear dimensionality reduction algorithm of Uniform Manifold Approximation and Projection (UMAP) was applied, which could divide or condense a group of patients into a series of distinct clusters, according to the given hallmarks or signatures. The hallmark gene sets of hypoxia include 200 genes and were downloaded from the Molecular Signatures Database (MSigDB version 6.0). Based on the clusters, two groups including “hypoxia**^low^**” and “hypoxia**^high^**” were identified to identify the hypoxia status. The limma algorithm was applied to identify differentially expressed genes (DEGs) between the two groups (16). Genes with a false discovery rate (FDR) adjusted p-value <0.0001 and an absolute value of log2 (fold change) >1 were considered as hypoxia-related DEGs.

### Identification of Immune Status and Immune-Related DEGs

To identify the immune status, the Estimation of Stromal and Immune cells in MAlignant Tumours using Expression data (ESTIMATE) algorithm was applied to identity the infiltration degree of immune cells and predict the immune status (17). Based on the immune status, patients were classified into two groups. Maximally selected rank statistics was applied by using an R package “survival”, and “survminer” to identify the optimal cutting point to divide patients. Based on the optimal cutting point, patients with high immune scores were attributed to “immune**^high^**” group and “immune**^low^**” group. The limma algorithm was applied to identify DEGs between the two groups. Genes with a FDR adjusted p-value <0.0001 and an absolute value of log2 (fold change) >1 were considered as immune-related DEGs.

To further identify the abundance of 22 immune cells, CIBERSORT is a deconvolution algorithm based on the gene expression data to resolve immune cell composition (18). Those with p <0.05 were included.

### Identification of Hypoxia-Immune-Related Prognostic DEGs

According to the above hypoxia and immune grouping, patients were divided into three groups, *i.e.*, hypoxia**^high^**/immune**^high^,** hypoxia**^low^**/immune**^low,^**and mix groups. The limma algorithm was applied to identify DEGs between “hypoxia**^high^**/immune**^high^** group and hypoxia**^low^**/immune**^low^** group. Genes with a FDR adjusted p-value <0.0001 and an absolute value of log2 (fold change) >1 were considered as hypoxia-immune-related DEGs. DEGs were then divided into protective and risk DEGs. The risk DEGs contained all EDGs highly expressed in hypoxia**^high^**/immune**^high^** group and the rest were protective DEGs. To obtain hypoxia-immune-related prognostic DEGs, univariate Cox analyses were further performed to screen all protective and risk DEGs. Those with a p <0.001 were considered as significant.

### Prognosis Prediction Model of IPF Based On Hypoxia-Immune-Related DEGs

The Least Absolute Shrinkage and Selection Operator (LASSO) is a kind of linear regression using shrinkage, which is applied to survival analysis with high-dimensional data (19). In this study, the LASSO Cox regression model was applied to select the optimal variables from all identified hypoxia-immune-related prognostic DEGs in the discovery cohort. Three-fold cross-validation and 1,000 iterations were conducted to reduce the potential instability of the results. The optimal tuning parameter *λ* was identified *via* 1-SE (standard error) criterion. Then we create the prognosis prediction model of IPF using the selected prognostic gene signature. For each patient, the risk score was the sum of the expression of the characteristic DEGs and the corresponding coefficients derived from the multivariate Cox regression model. According to the risk scores, the optimal cutting point was identified using the maximally selected rank method, and the prognosis prediction model of IPF was formed.

### Functional and Pathway Enrichment Analysis

Database for Annotation, Visualization and Integrated Discovery was used for Gene Ontology (GO) enrichment analysis (20). The risk DEGs of IPF patients were screened for functional enrichment. GO analysis was used to evaluate the degree of enrichment of the DEGs in biological processes, cellular components, and molecular functions. Those with p-value <0.05 and count (the number of enriched genes) ≥3 were considered as the cutoff criterion.

### Preliminary Validation of Clinical Specimens

Peripheral blood mononuclear cell (PBMC) and bronchoalveolar lavage fluid (BALF) were collected from 22 clinical samples, including 13 healthy controls, six with non-fibrotic pneumonia and three with pulmonary fibrosis. The clinical information such as age, gender, alveolar-arterial oxygen gradient (A-aDO2), and hospital day was shown in **Supplementary Materials**. The study was reviewed and approved by the institutional review board (Ethics Committee) of the 3rd Xiangya Hospital, Central South University (No. 21028).

Quantitative reverse transcriptase-PCR (qRT-PCR) was used to quantitative expression of key DEGs. Total RNA was extracted from the tissues using TRIzol Reagent (Thermo Fisher Scientific). PCR was performed using an Thermo Scientific PikoReal PCR cycler. The cycle threshold (CT) data were determined, and the mean CT was determined from triplicate PCRs. Relative gene expression was calculated with the equation 2^–ΔCT^.

Flow cytometry analysis was used to determine the proportion of immune cells. The cell suspension was counted and mixed with ACK Lysis Buffer (Thermo Fisher Scientific) to remove red blood cells. Then 1 × 10^6^ cells were resuspended in 100 µl staining buffer and incubated with monoclonal antibodies in dark for 15 min at 4°C. Our flow cytometric staining strategy consisted of the following fluorochrome-conjugated monoclonal antibodies: anti-CD3-Alexa-Flour700 (Biolegend), anti-CD4-eFlour450 (eBioscience), anti-CD45RA-APC-eFluor 780 (eBioscience), anti-CCR7-PerCP-eFluor 710 (eBioscience), anti-CD16-eFluor506 (eBioscience), anti-CD56-PE (eBioscience), anti-CD206- PE-Cyanine7 (eBioscience), anti-CD68-FITC (eBioscience), anti-CD107a-eFluor660 (eBioscience). CD3 and CD4 were used to identify T cells. CD3^+^CD4^+^CD45RA^−^CCR7^−^ cells were defined as activated CD4^+^ memory T cells. CD56 and CD16 were used to identify NK cells. CD107a^+^ NK cells were defined as activated NK cells. CD68^+^CD16^−^CD206^−^ cells were defined as M1-like macrophages and CD68^+^CD16^−^CD206^+^ cells were defined as M0-like macrophages. After washing and resuspending, samples were detected using BD FACSDiva software and performed using BD FACSCanto II.

### Statistical Analysis

All analyses were performed with R version 4.0.2 (www.r-project.org/) and the corresponding packages. UMAP algorithm was performed by using R package “umapr” for non-linear dimensionality reduction. Immune score was performed by using R package “estimate”. The Lasso Cox regression model was performed by using R package “glmnet”. Data were analyzed with standard statistical tests as appropriate. Multiple testing was adjusted by the FDR method. Multivariate Cox regression analysis was performed to identify optimal signatures. Flowjo V 10.62 was used to analyzed flow cytometric data. Original data from PCR and flow cytometry were presented as the mean ± standard deviation (SD) and were compared using Student’s t-test, Welch’s t-test or the Mann–Whitney U test, where appropriate. GraphPad Prism 7.0 (GraphPad Software Inc., La Jolla, CA, USA) was used to perform the statistical analyses. Values of p <0.05 were considered statistically significant.

**Figure 1:**
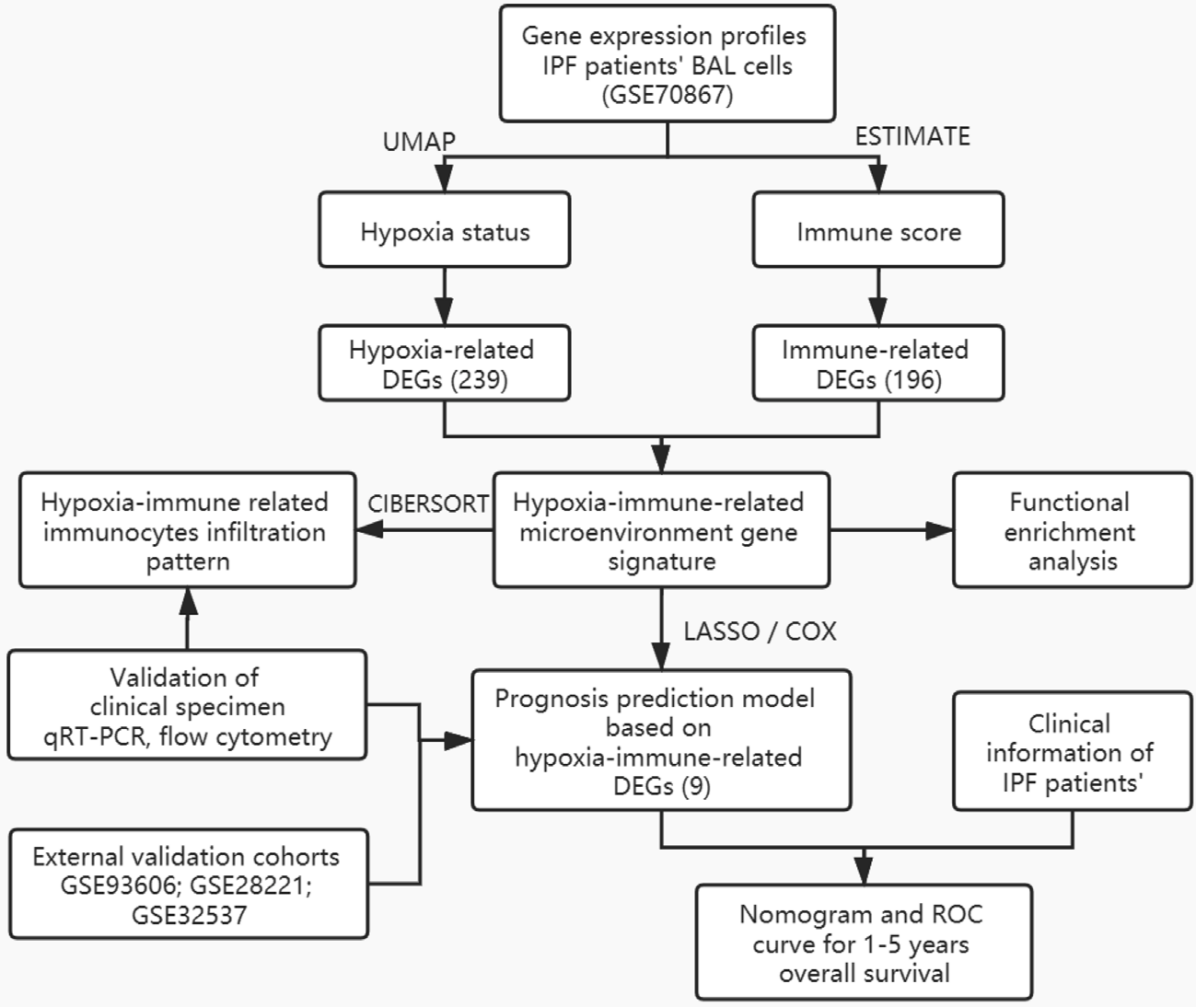
Flow chart of methodologies used in this study.

The general idea and methodologies used in this study were drawn as a flow chart (**Figure 1**).

## Results

### Hypoxia Status and Hypoxia-Related DEGs in IPF

**Figure 2:**
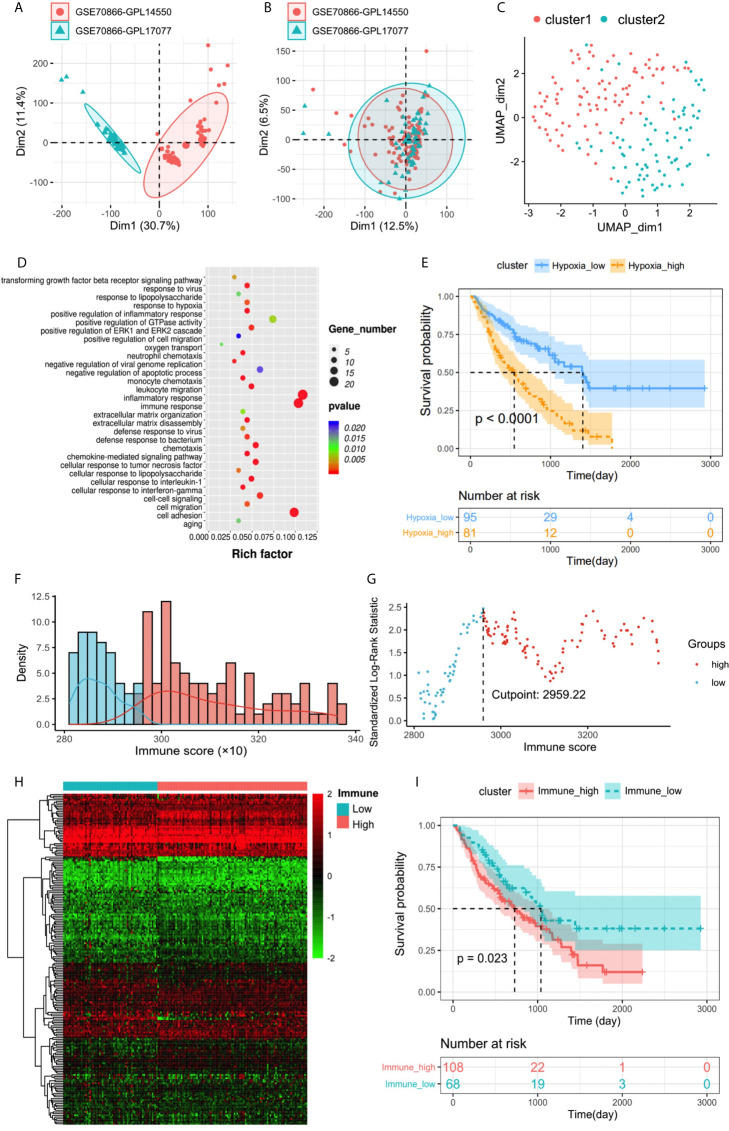
**(A, B)** Eliminating the batch effect between different sequencing platforms. **(A)** The PCA plot before elimination of batch effect, and **(B)** is the PCA plot after elimination. The distance of sample point clusters indicates that they come from different batches and sequencing platforms. While in B, after eliminating the batch effect, the difference in distance between batches is reduced. **(C)** UMAP clustering plot based on marker gene set of hypoxia. **(D)** Biological process functional enrichment analysis of differentially expressed genes between hypoxia**^high^** and hypoxia**^low^** groups. **(E)** Kaplan–Meier plot of overall survival in two clusters. **(F)** Histogram based on maximally selected rank grouping. **(G)** The cut-off point with the maximum standard log-rank statistic was marked with a vertical dashed line. **(H)** The differential gene expression profiles between hypoxia**^high^** and hypoxia**^low^** groups were visualized in heatmap. **(I)** Kaplan–Meier plot of overall survival between immune**^high^** and immune**^low^** groups.

The discovery cohort contained 176 IPF patients from the GEO databases. The batch effect was eliminated by sva package (**Figures 2A, B**). Patient clinical information is shown in **Table 1**. With the expression matrix constructed by 200 hypoxia marker genes from MSigDB, the non-linear dimensionality reduction algorithm UMAP was used to determine two clusters, and each patient is assigned to the nearest clusters (**Figure 2C**). Cluster 1 and Cluster 2 contained 95 and 81 patients respectively. Expression profiles were compared between the two clusters, and 239 DEGs related to hypoxia were obtained. Enrichment analysis showed overexpressed genes in Cluster 2 were enriched in “oxygen transport (GO:0015671)” and “response to hypoxia (GO:0001666)” (**Figure 2D**). This indicated that the level of hypoxia in Cluster2 was at high status. Thereby, the patients in Cluster1 and Cluster2 were determined as hypoxia**^low^** and hypoxia**^high^** groups. In addition, overexpressed genes in Cluster2 were also enriched in positive regulation of GTPase activity and cell adhesion. Patients’ clinical information of each cluster is shown in **Table 2**. The survival status of patients in different groups was further analyzed (**Figure 2E**). There was a significant difference in survival between two clusters (log rank test, p < 0.0001), and the prognosis of patients with a high level of hypoxia is worse. Among 239 DEGs, 232 DEGs were overexpressed in the hypoxia**^high^** cluster, which were regarded as hypoxia-associated risk DEGs. The other seven genes overexpressed in the hypoxia**^low^** cluster, which are regarded as hypoxia-associated protective DEGs. In a word, most of the hypoxia-related DEGs are regarded as risk factors.

**Table 1:** Basic information of IPF patients in discovery cohort.

Characteristics	Whole cohort (176)	Low risk (55)	High risk (121)
**Gender**			
Male	117 (0.665)	31 (0.564)	86 (0.711)
Female	59 (0.335)	24 (0.436)	35 (0.289)
**Age**			
≥65 years	86 (0.489)	30 (0.545)	56 (0.463)
<65 years	90 (0.511)	25 (0.455)	65 (0.537)
**UMAP clustering**			
Cluster1	95 (0.540)	47(0.855)	48 (0.397)
Cluster2	81 (0.460)	8 (0.145)	73 (0.603)
**Hypoxia status**			
High	81 (0.460)	8 (0.145)	73 (0.603)
Low	95 (0.540)	47(0.855)	48 (0.397)
**Immune status**			
High	108 (0.614)	21 (0.382)	87 (0.719)
Low	68 (0.386)	34 (0.618)	34 (0.281)
**Risk group**			
High	121 (0.688)	0	121 (1.000)
Low	55 (0.312)	55 (1.000)	0

**Table 2:** Basic information of IPF patients in different hypoxia-based clusters.

Characteristics	Whole cohort (176)	hypoxia^low^ (95)	hypoxia^high^ (81)
**Gender**			
Male	117 (0.665)	58 (0.610)	59 (0.728)
Female	59 (0.335)	37 (0.390)	22 (0.272)
**Age**			
≥65 years	86 (0.489)	48 (0.505)	38 (0.470)
<65 years	90 (0.511)	47 (0.495)	43 (0.530)

### Immune Status and Immune-Related DEGs in IPF

The immune score was calculated by ESTIMATE to identity the infiltration degree of immune cells. The optimal cutting point “2,959.22” was determined based on maximally selected rank statistics (**Figures 2F, G**). Then the immune**^high^** group and immune**^low^** group were divided, containing 108 and 68 patients respectively. Patients’ clinical information of each cluster is shown in **Table 3**. Further survival analysis showed a significant difference between two groups (log rank test, p < 0.05), and the survival of patients with a high level of immune infiltration is worse (**Figure 2I**). Therefore, high immune infiltration is also a risk factor for bad prognosis. This conclusion is also supported by the highly enriched immune-related pathways in the hypoxia**^high^** group with a poor prognosis. Expression profiles were compared between the two groups, and 196 DEGs related to immune status were obtained (**Figure 2H**). Among them, 191 genes were overexpressed in the immune**^high^** cluster, which were regarded as immune-associated risk DEGs. The other five genes overexpressed in the immune**^low^** cluster, which are regarded as immune-associated protective DEGs.

**Table 3:** Basic information of IPF patients in different immune-based clusters.

Characteristics	Whole cohort (176)	immune^low^ (68)	immune^high^ (108)
**Gender**			
Male	117 (0.665)	44 (0.647)	72 (0.667)
Female	59 (0.335)	24 (0.353)	36 (0.333)
**Age**			
≥65 years	86 (0.489)	38 (0.559)	48 (0.445)
<65 years	90 (0.511)	30 (0.441)	60 (0.555)

### Hypoxia–Immune-Related DEGs in IPF

**Figure 3:**
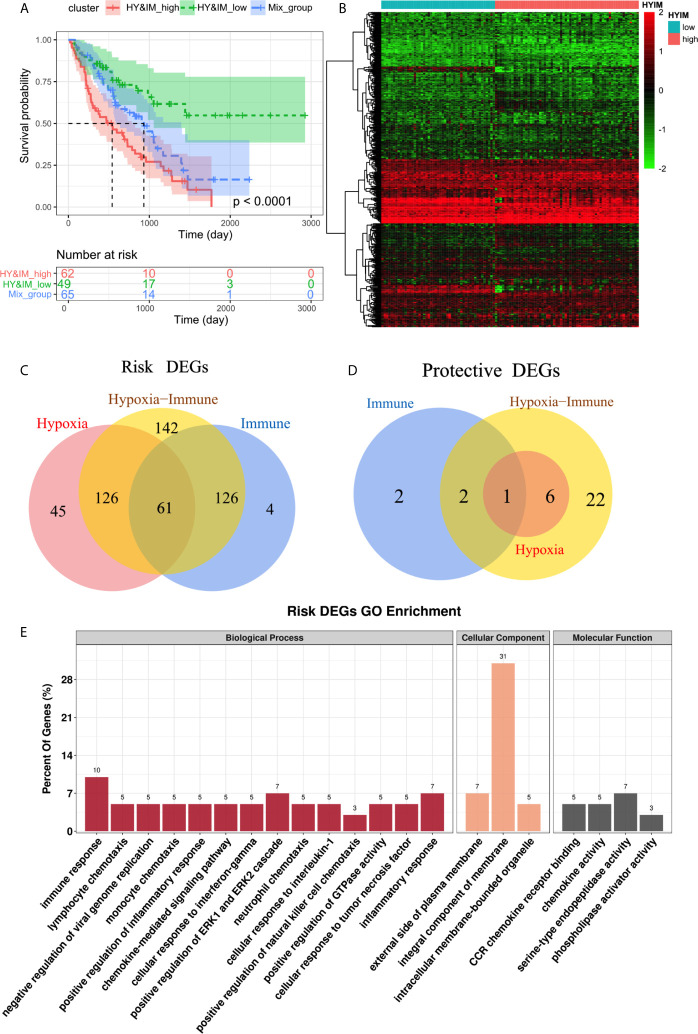
**(A)** Kaplan–Meier plot of overall survival between hypoxia**^high^**/immune**^high^**, hypoxia**^low^**/immune**^low^**, and mix groups. **(B)** Heatmap shows the differential gene expression profiles between hypoxia**^high^**/immune**^high^** and hypoxia**^low^**/immune**^low^** groups **(C,D)** Venn diagrams show the hypoxia-immune related risk DEGs (61) and protective DEG (1). € GO enrichment analysis of risk DEGs.

According to the above hypoxia and immune grouping, we further combined to form three groups: hypoxia**^high^**/immune**^high^,** hypoxia**^low^**/immune**^low^,** and mix groups. The survival status of patients in different groups was further analyzed (**Figure 3A**). There was a significant difference in survival among three groups (log rank test, p < 0.0001). Survival in mix group is at an intermediate level. As we expected, a high level of hypoxia and immune activity is the most dangerous factor while patients in group hypoxia**^low^**/immune**^low^** have the best prognosis.

The differential gene expression profiles between hypoxia**^high^**/immune**^high^** and hypoxia**^low^**/immune**^low^** groups were visualized in heatmap (**Figure 3B**). We further intersect the hypoxia-related DEGs and the immune-related DEGs to identify the hypoxia–immune-related DEGs in IPF. We obtained a total of 62 DEGs, of which 61 were highly expressed in hypoxia**^high^** and immune**^high^** groups, so they were defined as hypoxia-immune-related risk DEGs (**Figure 3C**). Correspondingly, the remaining DEG is defined as hypoxia-immune-related protective DEG (**Figure 3D**). The GO enrichment analysis showed that “immune response”, “inflammatory response”, and “positive regulation of ERK1/2 cascade” are main biological process (**Figure 3E**).

### Prognosis Prediction Model of IPF Based on Hypoxia-Immune-Related DEGs

**Figure 4:**
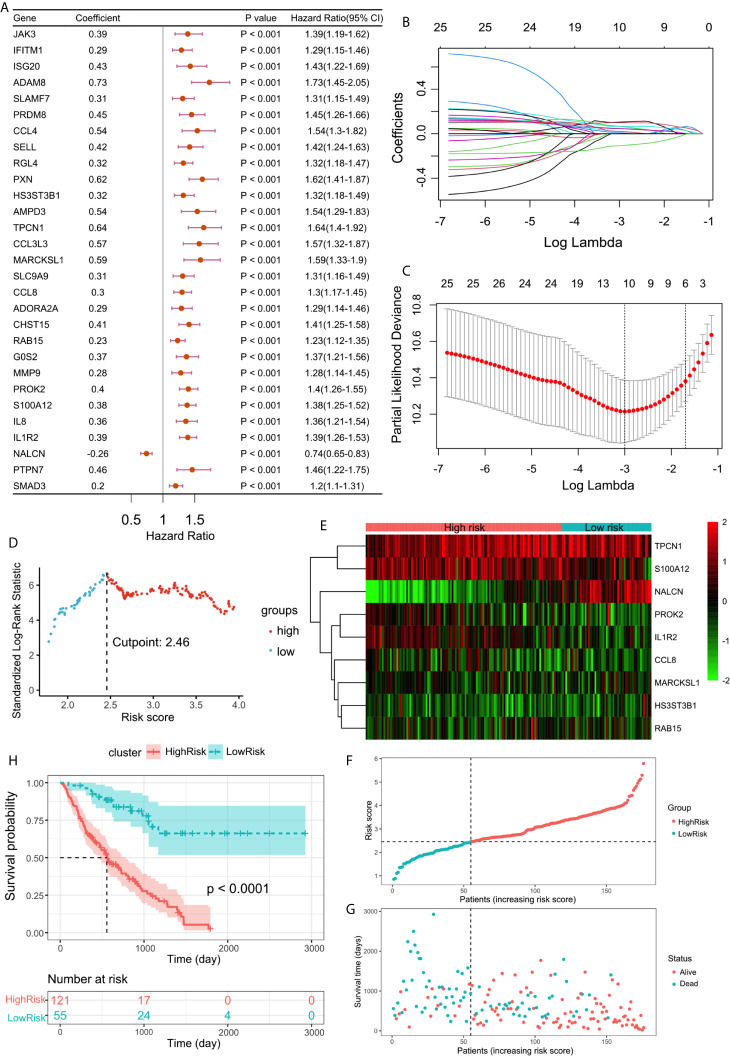
**(A)** Forest plot of 29 DEGs with P <0.001 by univariate Cox regression. **(B)** LASSO coefficient profiles of 29 screened DEGs. **(C)** Three-fold cross-validation of lasso analysis. Error bars represented the SE. The dotted vertical lines showed the optimal values. **(D)** The cut-off point with the maximum standard log-rank statistic was marked with a vertical dashed lines. **(E)** The expression profiles of DEGs involved in multivariate Cox regression model. **(F)** The distribution of patients with increased risk score in two groups. **(G)** Scatter plot showed the survival of patients with increased risk score. **(H)** Kaplan–Meier plot of overall survival between high-risk and low-risk groups.

To further determine the DEGs significantly related to the prognosis, we used univariate Cox analysis for screening and 29 DEGs with p <0.001 were retained (**Figure 4A**). Among them, one protective and 28 risk DEGs were included.

Using lasso regression method, nine optimal variables were obtained from the above 29 hypoxia-immune-prognostic-related DEGs (**Figures 4B, C**). Then we use the expression levels of nine characteristic DEGs and the corresponding coefficients derived from the multivariate Cox regression model to estimate the risk score for each patient: risk score = −0.13307 × expression of NALCN + 0.09893 × expression of IL1R2 + 0.06226 × expression of S100A12 + 0.06890 × expression of PROK2 + 0.04883 × expression of CCL8 + 0.05654 × expression of RAB15 + 0.10671 × expression of MARCKSL1 + 0.09986 × expression of TPCN1 + 0.05696 × expression of HS3ST3B1.

By calculating the risk score of each patient, we divided the patients into two groups through the maximally selected rank method: high-risk group and low-risk group (**Figure 4D**). The nine optimal DEG expression profiles between high-risk and low-risk groups were visualized in heatmap (**Figure 4E**). Survival analysis showed that there was a significant difference between the two groups (log rank test, p < 0.0001). Compared with the low-risk group, the prognosis of the high-risk group is significantly worse (**Figures 4F–H**).

### Hypoxia-Immune Related Immunocyte Infiltration Pattern

**Figure 5:**
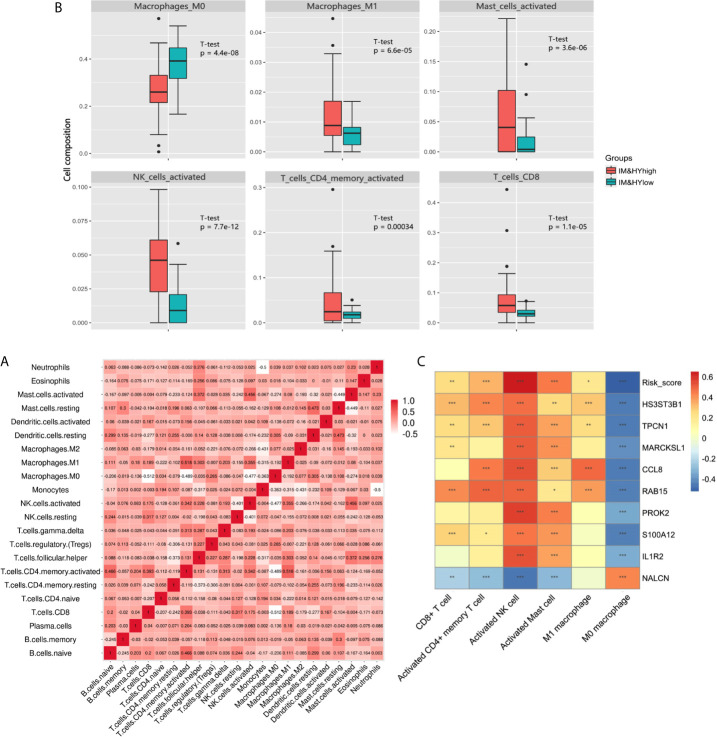
**(A)** Heatmap showed the correlation coefficient between different immune cells. **(B)** The box plot showed the significant difference of immune cells’ infiltration between two groups. **(C)** Heatmap showed the correlation coefficient between immune cells and DEGs involved in Cox model (*** means P < 0.01, ** means P < 0.05, and * means P < 0.1).

In addition, CIBERSORT was used to estimate the infiltration of 22 kinds of immune cells in the samples. Correlation analysis showed a general association in different immune cells (**Figure 5A**). Among them, the infiltration of six specific immune cells was significantly different in hypoxia**^high^**/immune**^high^** and hypoxia**^low^**/immune**^low^** group, that is, CD8+ T cell, activated CD4+ memory T cell, activated natural killer (NK) cell, activated mast cell, M0 macrophage, M1 macrophage (**Figure 5B**). Further correlation analysis showed the relationship between six specific immune cells and risk score. Among them, most infiltration degree is positively correlated with risk DEGs expression and risk score, but the infiltration of M0 cells was negatively correlated with the risk DEGs expression and risk score (**Figure 5C**). Among them, M0 macrophages and NK cells had the most significant correlation with key DEGs and risk score.

### Validation of the Prognostic Prediction Model in External Independent Cohorts

**Figure 6:**
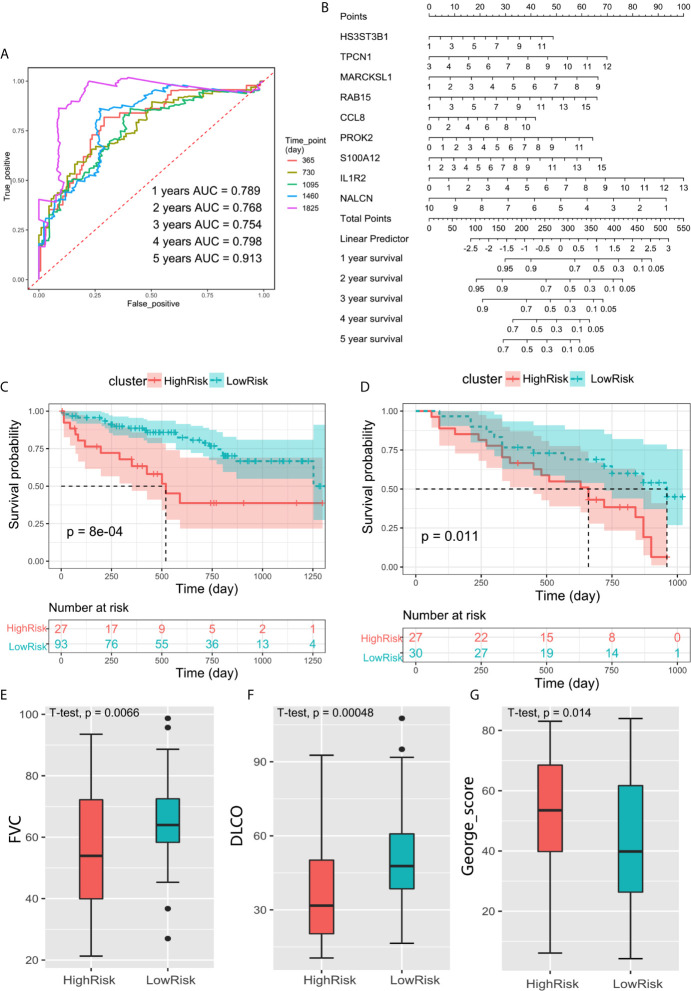
**(A)** ROC curve evaluated the predictive value of the model for the prognosis of patients in discovery cohort within 1–5 years. **(B)** The nomogram for 1–5 years overall survival based on Cox model. **(C)** Kaplan–Meier plot of overall survival between high-risk and low-risk groups in GSE28221 validation cohort. **(D)** Kaplan–Meier plot of overall survival between high-risk and low-risk groups in GSE93606 validation cohort. **(E–G)** The box plot showed the difference of FVC, DLCO, and George’s score between low-risk group and high-risk groups in GSE32537 validation cohort.

The ROC curve showed that the AUCs within 1–5 years were all greater than 0.75 in discovery cohort (**Figure 6A**). This suggested the evaluation model had a good predictive value for the prognosis of IPF patients. We also developed a nomogram for 1–5 years overall survival prediction based on Cox model (**Figure 6B**).

We further verify the above prediction method in external data sets” GSE93606”, “GSE28221”, and “GSE32537”. Patient clinical information is shown in **Table 4**. In each independent validation cohort, we divided the IPF patients into high-risk and low-risk groups based on the risk score. In GSE28221 and GSE93606 IPF cohorts, survival comparison showed that low-risk group had significantly better prognosis outcomes than high-risk group (**Figures 6C, D**). In addition, we focused on the clinicopathologic features of IPF in GSE32537 cohort. The patients in low-risk group generally had higher forced vital capacity (FVC) and carbon monoxide diffusing capacity (DLCO) (P < 0.05), which meant better lung function (**Figures 6E, F**). While the patients in high-risk group had higher St. George’s total score (P < 0.05), which suggested worse lung function and quality of life (**Figure 6G**).

**Table 4:** Basic information of IPF patients in validation cohort.

Characteristics	Whole cohort (296)	Low risk (182)	High risk (114)
**GSE93606**	Whole cohort (57)	Low risk (30)	High risk (27)
**Gender**			
Male	38 (0.667)	18 (0.600)	20 (0.741)
Female	19 (0.333)	12 (0.400)	7 (0.259)
**Age**			
≥65 years	35 (0.614)	18 (0.600)	17 (0.630)
<65 years	22 (0.386)	12 (0.400)	10 (0.370)
**GSE32537**	Whole cohort (119)	Low risk (59)	High risk (60)
Gender			
Male	42 (0.353)	25 (0.424)	17 (0.283)
Female	77 (0.647)	34 (0.576)	43 (0.717)
**Age**			
≥65 years	53 (0.445)	31 (0.525)	22 (0.367)
<65 years	66 (0.555)	28 (0.475)	38 (0.633)
**GSE28221**	Whole cohort (120)	Low risk (93)	High risk (27)
**Gender**			
Male	92 (0.767)	71 (0.763)	21 (0.778)
Female	28 (0.233)	22 (0.237)	6 (0.222)
**Age**			
≥65 years	76 (0.633)	55 (0.591)	21 (0.778)
<65 years	44 (0.367)	38 (0.409)	6 (0.222)

In a word, these results suggest that our prognostic prediction model is also of great significance based on peripheral whole blood, peripheral blood mononuclear cell, and lung tissue.

### Preliminary Validation of Clinical Specimens

**Figure 7:**
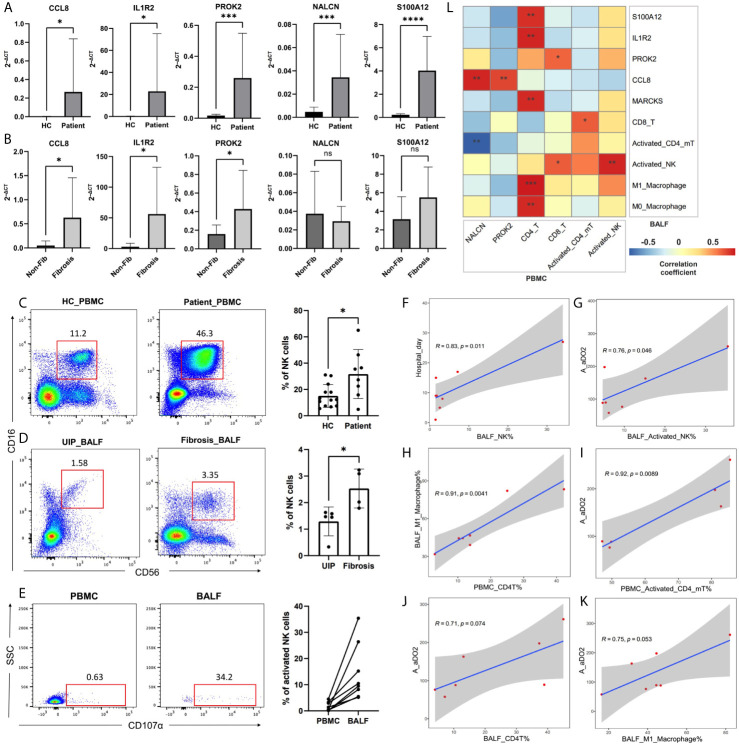
**(A, B)** The plots showed the results of qRT-PCR. Plot A showed the difference of DEGs expression in PBMC between healthy controls (HC) and patients. Plot B showed the difference of DEGs expression in PBMC between non-fibrotic pneumonia group (Non-Fib) and pulmonary fibrosis group (Fibrosis). **(C–E)** CD56 and CD16 were used to identify NK cells in flow cytometry. CD56^+^CD16^+^CD107a^+^ cells were defined as activated NK cells. Plot C showed the difference of NK% in PBMC between healthy controls (HC) and patients. Plot D showed the difference of NK% in BALF between usual interstitial pneumonitis (UIP, from Non-Fib group) and pulmonary fibrosis group (Fibrosis). **(F–K)** Pearson analysis showed the correlation between cell proportion and clinical features. **(L)** Pearson analysis showed the correlation between the results of BALF and PBMC. *, **, ***, **** respectively represent P values of t-test < 0.05, < 0.01, < 0.001, < 0.0001.

The results of qRT-PCR in PBMC suggested that DEGs of CCL8, IL1R2, NALCN, S100A12, and PROK2 were significantly different between the patients and healthy controls (**Figure 7A**). Among the patients, CCL8, IL1R2, and PROK2 were significantly up-expressed in fibrotic samples than non-fibrotic samples (**Figure 7B**). The PBMC results of flow cytometry showed that the proportion of NK cells was increased significantly in the patients than healthy controls (**Figure 7C**), and among the patients’ BALF, the proportion of NK cells was also increased significantly in fibrotic samples than in non-fibrotic ones (**Figure 7D**). The proportion of activated NK cells in BALF samples was significantly higher than those in PBMC samples, and it had the following characteristics: the increasing trend of peripheral blood to pulmonary bronchus (**Figure 7E**). The proportion of NK or activated NK cells’ infiltration in BALF was positively correlated with patients A-aDO2 and hospital day, suggesting that high NK infiltration is a risk factor for poor prognosis (**Figures 7F, G**). In addition, the flow cytometry showed that the increasing CD4+ T cells in peripheral blood might promote the macrophage infiltration in pulmonary bronchus and promoted their polarization to M1-like phenotype (**Figure 7H**). The proportion of CD4+ T, activated CD4+ memory T cells, and M1-like macrophages infiltration in BALF or PBMC was positively correlated with patients’ A-aDO2 (**Figures 7I–K**). **Figure 7L** showed the relationship between the results of BALF and PBMC. The detailed data of sample information and experimental results of BALF/PBMC were shown in the **Supplementary Materials**.

## Discussion

Since the prognosis of patients with IPF is poor, the importance of building a prognostic staging system for personalized treatment is self-evident. The prognostic staging system could divide the patients into several groups according to the given markers. In this study, we used the transcriptional profiles of the bronchoalveolar lavage fluid (BALF) to analyze the relationship between the level of biomarkers and the prognosis of patients. We found that both hypoxia and immune status were related to the survival and even respiratory function of patients with IPF. Furthermore, we established a new prognostic classifier including nine-gene signature for patients with IPF. It is effective in the prognosis of patients with IPF in the discovery cohort and three independent validation cohorts. These findings provide a new insight to the relationship between biomarkers in the lung milieu and the prognosis and stratification of patients with IPF.

Several articles reported the role of immune and hypoxia microenvironment in lung diseases (15). On one hand, immune dysregulation and inflammation are regarded as the basis of chronic lung diseases, including IPF (11). Bioinformatics analysis of RNA network and immune infiltration showed that immune cells were associated with the severity of IPF (21). Both innate and adaptive immunity were activated in fibrogenesis (22). On the other hand, hypoxia is common in lung disease. Hypoxia-inducible factor-1*α* (HIF-1*α*) is a key regulating factor in cell response to hypoxia, which has been found to participate in many lung diseases (23–25). In hypoxia, the activation of HIF-1*α* mediates glycolysis modification, angiogenesis, and other adaptive mechanism (26, 27). Hypoxia promoted the epithelia–mesenchymal transition (EMT) of alveolar epithelial cells (AECs) in IPF, and transforming growth factor *β* (TGF-*β*) also promoted EMT with increased lactic acid produced by metabolic modification (24, 28). Also, HIF-1*α* was found to be active in fibroblasts from IPF patients and induced myofibroblast differentiation with the existence of TGF-*β* (24, 26, 29, 30). It is worth mentioning that hypoxia facilitated proliferation and the secretion of proinflammatory cytokines in mast cells, and thus influenced fibrogenesis (31). In IPF, alveolar macrophages showed a perturbation of mitochondria homeostasis, including increased mitochondria reactive oxygen species (mtROS), in which HIF-1*α* may have participated (32). These findings were in accordance with the results. The result of infiltration of different types of immune cells showed that both innate immunity and adaptive immunity were activated in hypoxia**^high^**/immune**^high^** group, presenting poorer prognosis. In a word, hypoxia, as the inducement of immune activation, mediates chronic airway inflammation and leads to fibrosis. Then, cell and organ dysfunction caused by fibrosis aggravates the formation of hypoxic inflammatory microenvironment, which forms a feedback loop.

The result of CIBERSORT showed that the infiltration of M0 macrophage in hypoxia**^low^**/immune**^low^** group was higher than hypoxia**^high^**/immune**^high^** group. As inactive and naive macrophages, the low proportion of M0 macrophages in the low-risk group suggested a lower level of inflammatory activation (33). At the same time, the infiltration of CD8+ T cell, activated CD4+ memory T cell, activated NK cell, activated mast cell, and M1 macrophage in hypoxia**^high^**/immune**^high^** group was higher than that in hypoxia**^low^**/immune**^low^** group, presenting a higher level of inflammation. As was discussed above, the role of CD4+ T cell, mast cell, and M1 macrophage in fibrosis was widely reported. It is worth noting that M0 macrophage and NK cell had the most significant correlation with key hypoxia-immune DEGs and risk score. However, the role of CD8+ T cell, NK cell, and M0 macrophage in fibrosis has not been fully verified. These may provide a new idea to understand the characteristic immune cells in fibrotic infiltration.

Also, we found that hypoxia-immune DEGs were mainly risk DEGs, taking part in the integral component of membrane and immune response, and the protective DEG was a gene encoding voltage-independent, non-selective cation channel. The roles of these predictive signature genes have been reported previously in lung diseases. Interleukin-1 receptor 2 (IL-1R2) is an anti-inflammatory cytokine. The increase level of IL-1R2 has been reported to be associated with poor prognosis in lung cancer (34, 35), and the increase level of IL-1 was associated with the development of chronic obstructive pulmonary disease (COPD) (36, 37). Another risk gene S100A12 is a novel inflammatory disease biomarker in acute respiratory distress syndrome (ARDS) (38), interstitial lung disease (ILD) (39), and COPD (40). Moreover, C-C Motif Chemokine Ligand 8 (CCL8) is a kind of monocyte chemoattractant, regulating group 2 ILCs in lung inflammation (41). These results were in accordance with the results in this article that the overexpression of IL-1R2, S100A12, and ILC2s may be predictive for poor diagnosis of IPF patients. NALCN gene encodes a voltage-independent, non-selective cation channel. Other signature genes are rarely reported in lung diseases. In this study, combing hypoxia and immune status, we identified these signature genes to provide new insights into the prognosis of IPF.

In particular, in the independent external validations of this study, the prognostic prediction model was performed in peripheral whole blood, peripheral blood mononuclear cell, and lung tissue of IPF patients, and the outcomes were significant. The preliminary clinical specimen validation suggested the reliability of most conclusions, but there are still limitations, such as insufficient sample size. And because of the individual differences and other confounding factors, the results based on the existing database must have some deviation from the reality. Although these results provided more possibilities and a wider application of this predictive model in clinical setting, a well-designed and multi-center study is needed for further exploration.

## Conclusions

The immune and hypoxia status in alveolar molecular environment is associated with the prognosis of patients with IPF. The prognostic model based on several signature genes raised a new way to predict the progression and prognosis of IPF.

## Data Availability Statement

Publicly available datasets were analyzed in this study. This data can be found here: Gene Expression Omnibus (GEO, available at: https://www.ncbi.nlm.nih.gov/geo/) database (GSE70866).

## Ethics Statement

The studies involving human participants were reviewed and approved by the institutional review board (Ethics Committee) of the 3rd Xiangya Hospital, Central South University (No. 21028). The patients/participants provided their written informed consent to participate in this study.

## Author Contributions

The study was conceived and designed by QZhu. Statistical analyses were performed by XL and YC. Software package was prepared by QYZ and YD. Flow cytometry was performed by XL and HC. Manuscript was written by XL, HC, and QZhu. All authors contributed to the article and approved the submitted version.

## Funding

This study was supported by grants from the National Natural Science Foundation of China (81700658) and the Hunan Provincial Natural Science Foundation-Outstanding Youth Foundation (2020JJ3058).

## Conflict of Interest

The authors declare that the research was conducted in the absence of any commercial or financial relationships that could be construed as a potential conflict of interest.

## Supplementary Material

Supplementary Figure 1 Kaplan–Meier plot of overall survival between hypoxia^high^/immune^high^, hypoxia^low^/immune^low^, hypoxia^high^/immune^low^ and hypoxia^low^/immune^high^ groups.
