# Supplementary figures and images for: Investigation of a Hypoxia-Immune-Related Microenvironment Gene Signature and Prediction Model for Idiopathic Pulmonary Fibrosis
Source: Front Immunol. 2021 Jun 14;12:629854. doi: 10.3389/fimmu.2021.629854 (PMC8236709; doi:10.3389/fimmu.2021.629854)

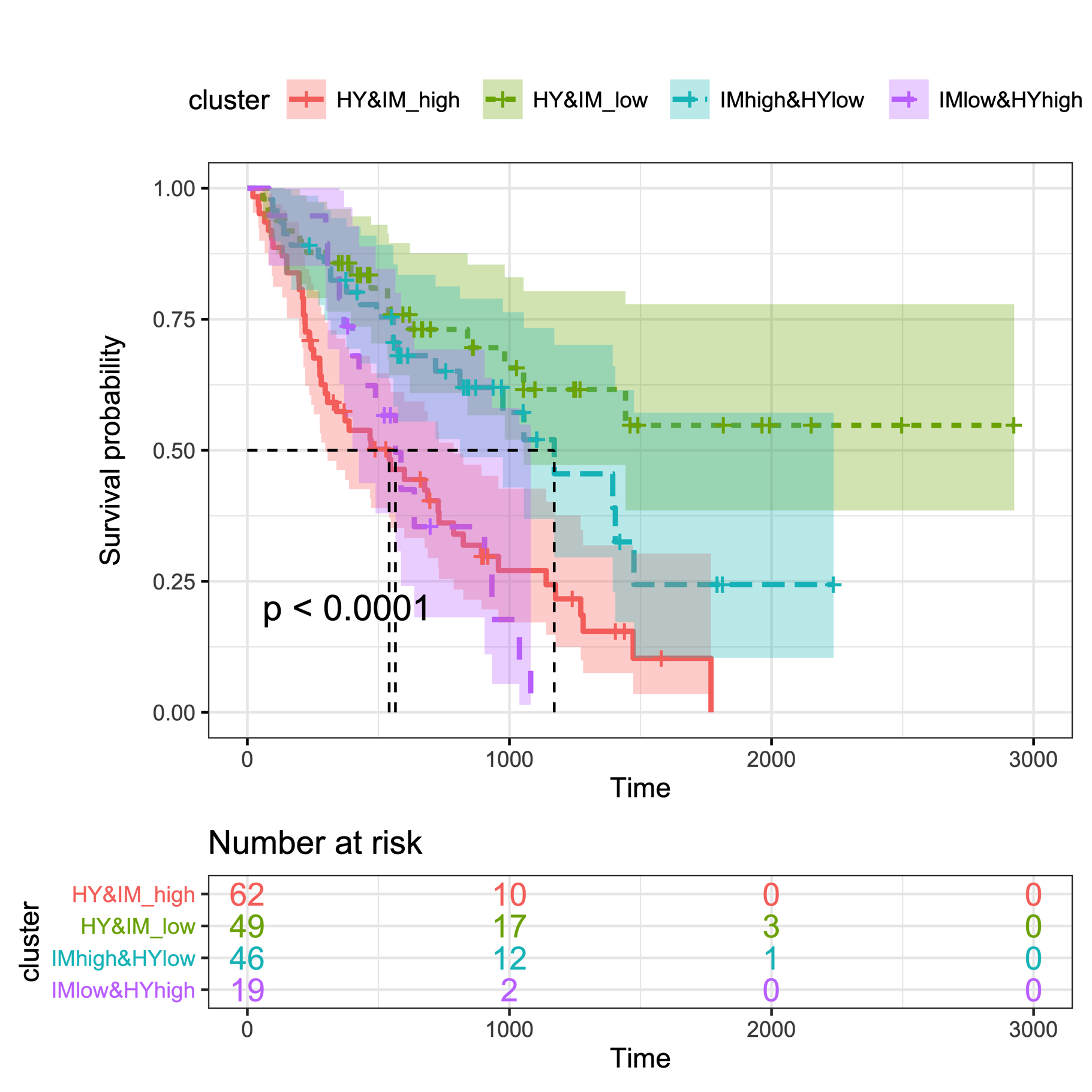

Supplement: Supplementary file 3 [file Image_1.tiff]
